# A new polymorph of 1-({[1,3-dihy­droxy-2-(hy­droxy­meth­yl)propan-2-yl]iminio}meth­yl)naphthalen-2-olate

**DOI:** 10.1107/S205698901501539X

**Published:** 2015-08-26

**Authors:** Ailing Guo, Shurong Zhang, Kun Wang, Ruitao Zhu

**Affiliations:** a School of Chinese Materia Medica, Shanxi University of Traditional Chinese Medicine, Taiyuan 030024, People’s Republic of China; b Department of Chemistry, Taiyuan Normal University, Taiyuan 030031, People’s Republic of China

**Keywords:** Schiff base, 2-hy­droxy-1-naphthaldehyde, O—H⋯O hydrogen bonding, N—H⋯O hydrogen bonding, crystal structure

## Abstract

The title compound, C_15_H_17_NO_4_, containing two mol­ecules in the asymmetric unit is a polymorph of the crystal structure published by Martínez *et al.* [(2011). *Eur. J. Org. Chem*. pp. 3137-3145] which at 120 K is monoclinic with one mol­ecule in the asymmetric unit. Both mol­ecules in the title compound are in the *trans* form. In the crystal, N—H⋯O and O—H⋯O hydrogen bonds connect mol­ecules, forming a two-dimensional network parallel to (001).

## Related literature   

For applications of Schiff bases, see: Weber *et al.* (2007 ▸); Chen *et al.* (2008 ▸); May *et al.*(2004 ▸). For background to the potential use of the title compound, see: Dong *et al.* (2014 ▸); Liu *et al.* (2014 ▸). For the structures of related Schiff bases derived from 2-hy­droxy­napthaldehyde, see: Wang *et al.* (2011 ▸); Kennedy *et al.* (2013 ▸); Abu-Dief *et al.* (2015 ▸). For the first polymorph, see: Martínez *et al.* (2011 ▸).
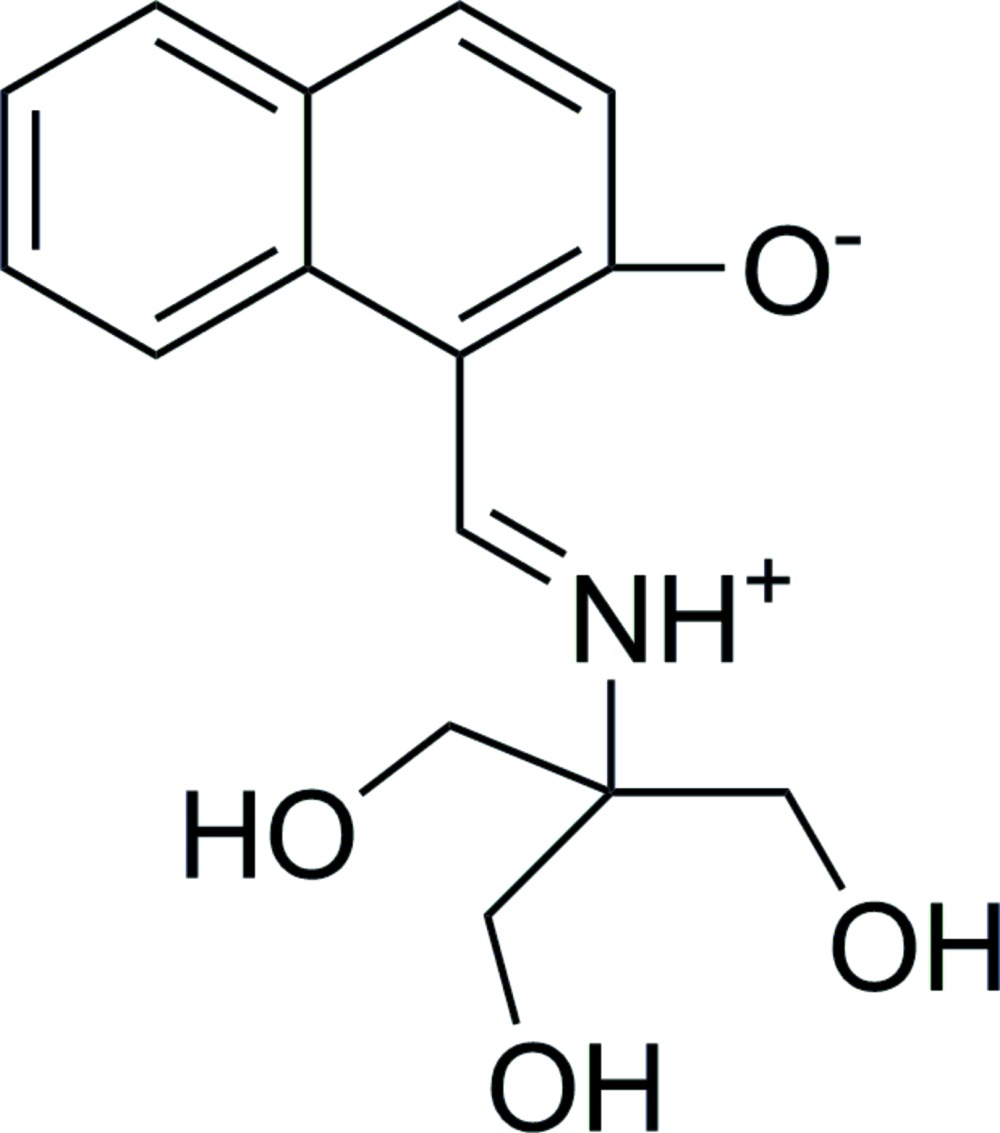


## Experimental   

### Crystal data  

C_15_H_17_NO_4_
*M* *_r_* = 275.30
Monoclinic, 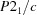

*a* = 9.3540 (8) Å
*b* = 10.0280 (9) Å
*c* = 29.036 (3) Å
β = 91.559 (1)°
*V* = 2722.6 (4) Å^3^
*Z* = 8
Mo *K*α radiation
μ = 0.10 mm^−1^
*T* = 293 K
0.49 × 0.45 × 0.44 mm

### Data collection  

Bruker SMART CCD area-detector diffractometer
Absorption correction: multi-scan (*SADABS*; Bruker, 2002 ▸) *T*_min_ = 0.954, *T*_max_ = 0.958
13224 measured reflections
4775 independent reflections
2778 reflections with *I* > 2σ(*I*)
*R*_int_ = 0.043

### Refinement  

*R*[*F* ^2^ > 2σ(*F* ^2^)] = 0.052
*wR*(*F* ^2^) = 0.137
*S* = 1.05
4775 reflections
368 parameters
H-atom parameters constrained
Δρ_max_ = 0.37 e Å^−3^
Δρ_min_ = −0.20 e Å^−3^

### 

Data collection: *SMART* (Bruker, 2002 ▸); cell refinement: *SAINT* (Bruker, 2002 ▸); data reduction: *SAINT*; program(s) used to solve structure: *SHELXS97* (Sheldrick, 2008 ▸); program(s) used to refine structure: *SHELXL97* (Sheldrick, 2008 ▸); molecular graphics: *SHELXTL* (Sheldrick, 2008 ▸) and *PLATON* (Spek, 2009 ▸); software used to prepare material for publication: *SHELXTL*.

## Supplementary Material

Crystal structure: contains datablock(s) I, New_Global_Publ_Block. DOI: 10.1107/S205698901501539X/lh5776sup1.cif

Structure factors: contains datablock(s) I. DOI: 10.1107/S205698901501539X/lh5776Isup2.hkl

**Fig. 1.**
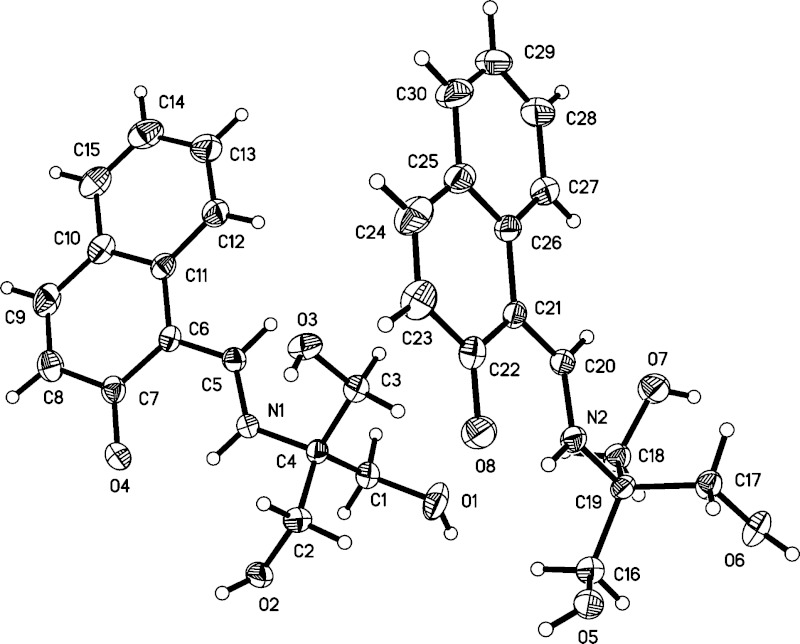
The molecular structure of the title compound with displacement ellipsoids drawn at the 30% probability level.

. DOI: 10.1107/S205698901501539X/lh5776fig1.tif
The mol­ecular structure of the title compound with displacement ellipsoids drawn at the 30% probability level.

**Fig. 2.**
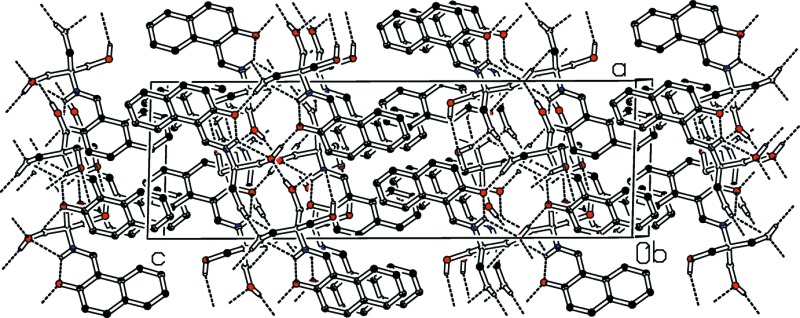
Part of the crystal structure with the hydrogen bonds drawn as dashed lines.

. DOI: 10.1107/S205698901501539X/lh5776fig2.tif
Part of the crystal structure with the hydrogen bonds drawn as dashed lines.

CCDC reference: 1419383

Additional supporting information:  crystallographic information; 3D view; checkCIF report

## Figures and Tables

**Table 1:** Hydrogen-bond geometry (, )

*D*H*A*	*D*H	H*A*	*D* *A*	*D*H*A*
N1H1O4	0.86	1.91	2.587(3)	135
N2H2O8	0.86	1.89	2.575(2)	135
O1H1*C*O5^i^	0.82	1.90	2.715(3)	172
O2H2*C*O8^ii^	0.82	1.77	2.589(3)	173
O3H3O6^iii^	0.82	1.91	2.706(3)	163
O5H5O4^iv^	0.82	1.84	2.650(2)	171
O6H6O2^v^	0.82	1.81	2.609(2)	163
O7H7O3^vi^	0.82	2.19	2.972(2)	159

Symmetry codes: (i) 
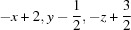
; (ii) 
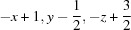
; (iii) 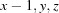; (iv) 
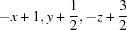
; (v) 
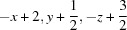
; (vi) 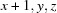.

## supplementary crystallographic information

### S1. Structural commentary

Schiff bases have been receiving considerable attention for many years, mainly due to their importance as ligands in metal complexes with special magnetic (Weber *et al.*, 2007) and selective fluorescence sensor (Dong *et al.*, 2014; Liu *et al.* , 2014), catalytic (Chen *et al.*, 2008) and biological properties (May *et al.*, 2004).

As a part of our studies on the synthesis and structural properties of Schiff bases with naphthaldehyde and methyl­amine, we have determined the structure of the title compound (Fig. 1). Some examples of related structures already appear in the literature (Wang *et al.*, 2011; Kennedy *et al.*, 2013; Abu-Dief *et al.*, 2015). The structure of the title compound contains two molecules in the asymmetric unit (Fig. 1) in contrast to the polymorph in which there is a single molecule (Martínez *et al.*, 2011). Both molecules in the title compound are in the *trans* form. In the crystal, N—H···O and O—H···O hydrogen bonds connect molecules forming a two-dimensional network parallel to (001) (Fig. 2).

### S2. Synthesis and crystallization

An ethanol solution (10 mL) of tris­(hy­droxy­methyl)­amino­methane (tris­, 0.1 mol, 0.1211g) was added to another ethanol (10mL) containing 2-hy­droxy-1-naphthaldehyde (0.1 mol, 0.1728 g), Then the solution was refluxed for 2 h and cooled to room temperature. The mixture was filtered and dried under vacuum. The title compound was crystallized as block crystals from a solution of ethanol by slow evaporation.

### S3. Refinement details

All H atoms were visible in differnce Fourier maps and the presence of those bonded bonded to N1 and N2 confirm the enolate form. Utimately, all H atoms were placed in calculated positions with C—H = 0.93–0.97Å, N—H = 0.86Å and O—H = 0.82Å and were included in the refinment in a riding-motion approximation with U_iso_(H)=1.2U_eq_(C,N) and U_iso_(H)=1.5U_eq_(O).

### Crystal data

**Table:**

C_15_H_17_NO_4_	*F*(000) = 1168
*M**_r_* = 275.30	*D*_x_ = 1.343 Mg m^−^^3^
Monoclinic, *P*2_1_/*c*	Mo *K*α radiation, λ = 0.71073 Å
Hall symbol: -P 2ybc	Cell parameters from 3152 reflections
*a* = 9.3540 (8) Å	θ = 2.5–25.6°
*b* = 10.0280 (9) Å	µ = 0.10 mm^−^^1^
*c* = 29.036 (3) Å	*T* = 293 K
β = 91.559 (1)°	Block, colorless
*V* = 2722.6 (4) Å^3^	0.49 × 0.45 × 0.44 mm
*Z* = 8	

### Data collection

**Table:**

Bruker SMART CCD area-detector diffractometer	4775 independent reflections
Radiation source: fine-focus sealed tube	2778 reflections with *I* > 2σ(*I*)
Graphite monochromator	*R*_int_ = 0.043
φ and ω scans	θ_max_ = 25.0°, θ_min_ = 2.5°
Absorption correction: multi-scan (*SADABS*; Bruker, 2002)	*h* = −11→11
*T*_min_ = 0.954, *T*_max_ = 0.958	*k* = −10→11
13224 measured reflections	*l* = −34→24

### Refinement

**Table:**

Refinement on *F*^2^	Secondary atom site location: difference Fourier map
Least-squares matrix: full	Hydrogen site location: inferred from neighbouring sites
*R*[*F*^2^ > 2σ(*F*^2^)] = 0.052	H-atom parameters constrained
*wR*(*F*^2^) = 0.137	*w* = 1/[σ^2^(*F*_o_^2^) + (0.0453*P*)^2^ + 1.3323*P*] where *P* = (*F*_o_^2^ + 2*F*_c_^2^)/3
*S* = 1.05	(Δ/σ)_max_ < 0.001
4775 reflections	Δρ_max_ = 0.37 e Å^−^^3^
368 parameters	Δρ_min_ = −0.19 e Å^−^^3^
0 restraints	Extinction correction: *SHELXL*, Fc^*^=kFc[1+0.001xFc^2^λ^3^/sin(2θ)]^-1/4^
Primary atom site location: structure-invariant direct methods	Extinction coefficient: 0.0078 (7)

### Special details

**Table:**

Geometry. All e.s.d.'s (except the e.s.d. in the dihedral angle between two l.s. planes) are estimated using the full covariance matrix. The cell e.s.d.'s are taken into account individually in the estimation of e.s.d.'s in distances, angles and torsion angles; correlations between e.s.d.'s in cell parameters are only used when they are defined by crystal symmetry. An approximate (isotropic) treatment of cell e.s.d.'s is used for estimating e.s.d.'s involving l.s. planes.
Refinement. Refinement of *F*^2^ against ALL reflections. The weighted *R*-factor *wR* and goodness of fit *S* are based on *F*^2^, conventional *R*-factors *R* are based on *F*, with *F* set to zero for negative *F*^2^. The threshold expression of *F*^2^ > σ(*F*^2^) is used only for calculating *R*-factors(gt) *etc*. and is not relevant to the choice of reflections for refinement. *R*-factors based on *F*^2^ are statistically about twice as large as those based on *F*, and *R*- factors based on ALL data will be even larger.

### Fractional atomic coordinates and isotropic or equivalent isotropic displacement parameters (Å^2^)

**Table:**

	*x*	*y*	*z*	*U*_iso_*/*U*_eq_	
N1	0.4266 (2)	0.5340 (2)	0.65750 (7)	0.0368 (5)	
H1	0.3778	0.4957	0.6784	0.044*	
N2	0.9319 (2)	0.9508 (2)	0.67169 (7)	0.0368 (5)	
H2	0.8792	0.9830	0.6928	0.044*	
O1	0.80392 (19)	0.5859 (2)	0.68816 (8)	0.0621 (6)	
H1C	0.8582	0.5347	0.7020	0.093*	
O2	0.49270 (19)	0.54635 (19)	0.74996 (6)	0.0468 (5)	
H2C	0.4247	0.5638	0.7662	0.070*	
O3	0.4541 (2)	0.8049 (2)	0.63099 (6)	0.0501 (5)	
H3	0.4281	0.8446	0.6540	0.075*	
O4	0.20984 (19)	0.3874 (2)	0.67796 (6)	0.0510 (5)	
O5	0.9988 (2)	0.93735 (19)	0.76456 (6)	0.0470 (5)	
H5	0.9340	0.9140	0.7812	0.071*	
O6	1.31332 (18)	0.91941 (18)	0.70090 (7)	0.0493 (5)	
H6	1.3611	0.9706	0.7171	0.074*	
O7	1.1405 (2)	0.7931 (2)	0.61213 (6)	0.0560 (6)	
H7	1.2275	0.7836	0.6111	0.084*	
O8	0.71162 (19)	1.0887 (2)	0.69349 (7)	0.0553 (6)	
C1	0.6809 (3)	0.5160 (3)	0.67280 (9)	0.0414 (7)	
H1A	0.6645	0.4406	0.6929	0.050*	
H1B	0.6943	0.4826	0.6419	0.050*	
C2	0.5234 (3)	0.6581 (3)	0.72181 (8)	0.0409 (7)	
H2A	0.6065	0.7050	0.7343	0.049*	
H2B	0.4430	0.7193	0.7212	0.049*	
C3	0.5793 (3)	0.7303 (3)	0.64202 (9)	0.0401 (7)	
H3A	0.6485	0.7884	0.6573	0.048*	
H3B	0.6206	0.6993	0.6137	0.048*	
C4	0.5521 (2)	0.6100 (3)	0.67308 (8)	0.0327 (6)	
C5	0.3813 (3)	0.5185 (3)	0.61482 (9)	0.0352 (6)	
H5A	0.4329	0.5604	0.5920	0.042*	
C6	0.2609 (2)	0.4436 (2)	0.60076 (9)	0.0341 (6)	
C7	0.1775 (3)	0.3805 (3)	0.63474 (10)	0.0403 (7)	
C8	0.0531 (3)	0.3070 (3)	0.61916 (11)	0.0558 (8)	
H8A	−0.0056	0.2676	0.6406	0.067*	
C9	0.0206 (3)	0.2948 (3)	0.57391 (12)	0.0593 (9)	
H9	−0.0601	0.2458	0.5652	0.071*	
C10	0.1033 (3)	0.3528 (3)	0.53867 (10)	0.0465 (7)	
C11	0.2235 (3)	0.4311 (3)	0.55185 (9)	0.0386 (6)	
C12	0.2988 (3)	0.4917 (3)	0.51637 (9)	0.0521 (8)	
H12	0.3777	0.5446	0.5239	0.063*	
C13	0.2595 (3)	0.4752 (4)	0.47095 (11)	0.0626 (9)	
H13	0.3120	0.5169	0.4483	0.075*	
C14	0.1425 (4)	0.3972 (3)	0.45827 (12)	0.0653 (9)	
H14	0.1170	0.3856	0.4274	0.078*	
C15	0.0658 (3)	0.3381 (3)	0.49156 (12)	0.0584 (9)	
H15	−0.0134	0.2867	0.4831	0.070*	
C16	1.0373 (3)	0.8292 (3)	0.73559 (9)	0.0427 (7)	
H16A	0.9611	0.7635	0.7346	0.051*	
H16B	1.1231	0.7866	0.7480	0.051*	
C17	1.1832 (2)	0.9830 (3)	0.68673 (9)	0.0389 (7)	
H17A	1.1919	1.0195	0.6560	0.047*	
H17B	1.1621	1.0556	0.7076	0.047*	
C18	1.0944 (3)	0.7591 (3)	0.65660 (9)	0.0421 (7)	
H18A	1.1673	0.7048	0.6718	0.051*	
H18B	1.0083	0.7056	0.6535	0.051*	
C19	1.0635 (2)	0.8799 (3)	0.68711 (8)	0.0340 (6)	
C20	0.8883 (3)	0.9693 (3)	0.62907 (8)	0.0343 (6)	
H20	0.9424	0.9323	0.6059	0.041*	
C21	0.7644 (2)	1.0416 (2)	0.61581 (8)	0.0332 (6)	
C22	0.6788 (3)	1.0992 (3)	0.65039 (10)	0.0399 (7)	
C23	0.5528 (3)	1.1703 (3)	0.63563 (11)	0.0544 (8)	
H23	0.4930	1.2061	0.6575	0.065*	
C24	0.5195 (3)	1.1862 (3)	0.59069 (12)	0.0601 (9)	
H24	0.4369	1.2330	0.5825	0.072*	
C25	0.6046 (3)	1.1347 (3)	0.55519 (10)	0.0468 (7)	
C26	0.7267 (3)	1.0578 (3)	0.56717 (9)	0.0379 (6)	
C27	0.8045 (3)	1.0038 (3)	0.53105 (9)	0.0499 (8)	
H27	0.8845	0.9517	0.5379	0.060*	
C28	0.7657 (4)	1.0257 (4)	0.48594 (10)	0.0666 (10)	
H28	0.8193	0.9881	0.4627	0.080*	
C29	0.6471 (4)	1.1036 (4)	0.47439 (12)	0.0718 (11)	
H29	0.6221	1.1195	0.4437	0.086*	
C30	0.5683 (4)	1.1560 (3)	0.50842 (12)	0.0660 (10)	
H30	0.4883	1.2072	0.5007	0.079*	

### Atomic displacement parameters (Å^2^)

**Table:**

	*U*^11^	*U*^22^	*U*^33^	*U*^12^	*U*^13^	*U*^23^
N1	0.0296 (11)	0.0475 (14)	0.0333 (13)	−0.0042 (10)	0.0018 (9)	0.0056 (10)
N2	0.0278 (11)	0.0511 (14)	0.0316 (12)	0.0033 (10)	0.0016 (9)	−0.0024 (10)
O1	0.0331 (11)	0.0564 (14)	0.0959 (18)	−0.0041 (10)	−0.0162 (11)	0.0145 (12)
O2	0.0380 (11)	0.0629 (13)	0.0397 (11)	0.0065 (9)	0.0037 (8)	0.0169 (10)
O3	0.0546 (12)	0.0534 (13)	0.0419 (11)	0.0079 (10)	−0.0087 (9)	0.0062 (10)
O4	0.0441 (11)	0.0665 (14)	0.0426 (12)	−0.0097 (10)	0.0063 (9)	0.0055 (10)
O5	0.0437 (11)	0.0620 (13)	0.0356 (11)	−0.0067 (10)	0.0048 (8)	−0.0086 (10)
O6	0.0334 (10)	0.0512 (13)	0.0626 (14)	0.0027 (9)	−0.0113 (9)	−0.0158 (10)
O7	0.0493 (12)	0.0768 (15)	0.0420 (12)	0.0052 (12)	0.0060 (9)	−0.0067 (11)
O8	0.0439 (12)	0.0816 (16)	0.0407 (12)	0.0112 (11)	0.0049 (9)	−0.0078 (11)
C1	0.0322 (15)	0.0486 (18)	0.0434 (16)	−0.0010 (13)	0.0009 (12)	0.0029 (13)
C2	0.0423 (15)	0.0474 (17)	0.0329 (15)	0.0007 (13)	−0.0015 (12)	0.0015 (13)
C3	0.0341 (14)	0.0450 (17)	0.0411 (16)	−0.0004 (13)	−0.0006 (12)	0.0030 (13)
C4	0.0270 (13)	0.0352 (15)	0.0357 (15)	−0.0040 (11)	−0.0013 (11)	0.0055 (12)
C5	0.0322 (14)	0.0388 (16)	0.0346 (15)	0.0034 (12)	0.0036 (11)	0.0015 (12)
C6	0.0280 (13)	0.0326 (15)	0.0415 (16)	0.0011 (11)	−0.0002 (11)	−0.0022 (12)
C7	0.0343 (15)	0.0380 (16)	0.0486 (18)	0.0026 (12)	0.0038 (13)	0.0031 (13)
C8	0.0478 (18)	0.055 (2)	0.064 (2)	−0.0175 (15)	0.0032 (16)	0.0070 (17)
C9	0.0485 (18)	0.051 (2)	0.078 (3)	−0.0189 (15)	−0.0109 (17)	−0.0016 (18)
C10	0.0438 (17)	0.0383 (17)	0.057 (2)	0.0016 (14)	−0.0068 (14)	−0.0080 (14)
C11	0.0339 (14)	0.0388 (16)	0.0429 (16)	0.0079 (13)	−0.0013 (12)	−0.0037 (13)
C12	0.0437 (17)	0.070 (2)	0.0425 (18)	−0.0051 (15)	−0.0020 (14)	−0.0053 (16)
C13	0.061 (2)	0.083 (3)	0.0440 (19)	0.0016 (19)	−0.0021 (16)	−0.0041 (18)
C14	0.073 (2)	0.074 (2)	0.047 (2)	0.013 (2)	−0.0115 (18)	−0.0167 (18)
C15	0.059 (2)	0.050 (2)	0.065 (2)	0.0017 (16)	−0.0177 (17)	−0.0164 (17)
C16	0.0406 (15)	0.0502 (18)	0.0372 (16)	−0.0016 (14)	0.0004 (12)	0.0007 (14)
C17	0.0316 (14)	0.0442 (17)	0.0409 (16)	0.0006 (12)	−0.0007 (12)	−0.0027 (13)
C18	0.0390 (15)	0.0477 (18)	0.0397 (17)	0.0002 (13)	0.0001 (12)	−0.0054 (13)
C19	0.0274 (13)	0.0444 (16)	0.0299 (14)	0.0012 (12)	−0.0031 (11)	−0.0012 (12)
C20	0.0313 (14)	0.0426 (16)	0.0291 (14)	−0.0034 (12)	0.0022 (11)	−0.0030 (12)
C21	0.0271 (13)	0.0342 (15)	0.0381 (15)	−0.0039 (11)	−0.0024 (11)	0.0023 (12)
C22	0.0313 (14)	0.0414 (17)	0.0470 (18)	−0.0006 (12)	0.0020 (12)	−0.0042 (13)
C23	0.0475 (18)	0.0482 (19)	0.067 (2)	0.0123 (15)	0.0002 (16)	−0.0088 (16)
C24	0.0533 (19)	0.0450 (19)	0.081 (3)	0.0147 (15)	−0.0151 (18)	0.0043 (17)
C25	0.0475 (17)	0.0376 (17)	0.0546 (19)	−0.0048 (14)	−0.0116 (14)	0.0112 (14)
C26	0.0382 (15)	0.0371 (16)	0.0380 (16)	−0.0084 (13)	−0.0059 (12)	0.0049 (13)
C27	0.0432 (17)	0.066 (2)	0.0402 (17)	−0.0057 (15)	−0.0022 (14)	0.0035 (15)
C28	0.068 (2)	0.094 (3)	0.0384 (18)	−0.017 (2)	−0.0031 (16)	0.0068 (18)
C29	0.080 (3)	0.089 (3)	0.046 (2)	−0.026 (2)	−0.0203 (19)	0.028 (2)
C30	0.065 (2)	0.061 (2)	0.070 (2)	−0.0049 (18)	−0.0240 (19)	0.0281 (19)

### Geometric parameters (Å, º)

**Table:**

N1—C5	1.308 (3)	C10—C15	1.411 (4)
N1—C4	1.461 (3)	C10—C11	1.415 (4)
N1—H1	0.8600	C11—C12	1.402 (4)
N2—C20	1.306 (3)	C12—C13	1.369 (4)
N2—C19	1.480 (3)	C12—H12	0.9300
N2—H2	0.8600	C13—C14	1.387 (4)
O1—C1	1.409 (3)	C13—H13	0.9300
O1—H1C	0.8200	C14—C15	1.356 (4)
O2—C2	1.421 (3)	C14—H14	0.9300
O2—H2C	0.8200	C15—H15	0.9300
O3—C3	1.419 (3)	C16—C19	1.523 (3)
O3—H3	0.8200	C16—H16A	0.9700
O4—C7	1.285 (3)	C16—H16B	0.9700
O5—C16	1.425 (3)	C17—C19	1.525 (3)
O5—H5	0.8200	C17—H17A	0.9700
O6—C17	1.425 (3)	C17—H17B	0.9700
O6—H6	0.8200	C18—C19	1.532 (3)
O7—C18	1.414 (3)	C18—H18A	0.9700
O7—H7	0.8200	C18—H18B	0.9700
O8—C22	1.285 (3)	C20—C21	1.412 (3)
C1—C4	1.530 (3)	C20—H20	0.9300
C1—H1A	0.9700	C21—C22	1.423 (3)
C1—H1B	0.9700	C21—C26	1.455 (3)
C2—C4	1.526 (3)	C22—C23	1.433 (4)
C2—H2A	0.9700	C23—C24	1.343 (4)
C2—H2B	0.9700	C23—H23	0.9300
C3—C4	1.532 (3)	C24—C25	1.417 (4)
C3—H3A	0.9700	C24—H24	0.9300
C3—H3B	0.9700	C25—C30	1.407 (4)
C5—C6	1.405 (3)	C25—C26	1.414 (4)
C5—H5A	0.9300	C26—C27	1.401 (4)
C6—C7	1.423 (3)	C27—C28	1.367 (4)
C6—C11	1.459 (3)	C27—H27	0.9300
C7—C8	1.440 (4)	C28—C29	1.391 (5)
C8—C9	1.346 (4)	C28—H28	0.9300
C8—H8A	0.9300	C29—C30	1.355 (5)
C9—C10	1.424 (4)	C29—H29	0.9300
C9—H9	0.9300	C30—H30	0.9300
			
C5—N1—C4	126.3 (2)	C15—C14—C13	119.1 (3)
C5—N1—H1	116.9	C15—C14—H14	120.4
C4—N1—H1	116.9	C13—C14—H14	120.4
C20—N2—C19	126.2 (2)	C14—C15—C10	121.5 (3)
C20—N2—H2	116.9	C14—C15—H15	119.2
C19—N2—H2	116.9	C10—C15—H15	119.2
C1—O1—H1C	109.5	O5—C16—C19	110.0 (2)
C2—O2—H2C	109.5	O5—C16—H16A	109.7
C3—O3—H3	109.5	C19—C16—H16A	109.7
C16—O5—H5	109.5	O5—C16—H16B	109.7
C17—O6—H6	109.5	C19—C16—H16B	109.7
C18—O7—H7	109.5	H16A—C16—H16B	108.2
O1—C1—C4	109.2 (2)	O6—C17—C19	108.4 (2)
O1—C1—H1A	109.8	O6—C17—H17A	110.0
C4—C1—H1A	109.8	C19—C17—H17A	110.0
O1—C1—H1B	109.8	O6—C17—H17B	110.0
C4—C1—H1B	109.8	C19—C17—H17B	110.0
H1A—C1—H1B	108.3	H17A—C17—H17B	108.4
O2—C2—C4	109.1 (2)	O7—C18—C19	113.9 (2)
O2—C2—H2A	109.9	O7—C18—H18A	108.8
C4—C2—H2A	109.9	C19—C18—H18A	108.8
O2—C2—H2B	109.9	O7—C18—H18B	108.8
C4—C2—H2B	109.9	C19—C18—H18B	108.8
H2A—C2—H2B	108.3	H18A—C18—H18B	107.7
O3—C3—C4	113.5 (2)	N2—C19—C16	106.65 (19)
O3—C3—H3A	108.9	N2—C19—C17	106.0 (2)
C4—C3—H3A	108.9	C16—C19—C17	111.7 (2)
O3—C3—H3B	108.9	N2—C19—C18	111.9 (2)
C4—C3—H3B	108.9	C16—C19—C18	107.9 (2)
H3A—C3—H3B	107.7	C17—C19—C18	112.5 (2)
N1—C4—C2	106.98 (19)	N2—C20—C21	124.3 (2)
N1—C4—C1	107.7 (2)	N2—C20—H20	117.8
C2—C4—C1	111.0 (2)	C21—C20—H20	117.8
N1—C4—C3	111.9 (2)	C20—C21—C22	119.3 (2)
C2—C4—C3	109.5 (2)	C20—C21—C26	119.8 (2)
C1—C4—C3	109.8 (2)	C22—C21—C26	120.9 (2)
N1—C5—C6	125.1 (2)	O8—C22—C21	122.0 (2)
N1—C5—H5A	117.5	O8—C22—C23	120.3 (2)
C6—C5—H5A	117.5	C21—C22—C23	117.7 (3)
C5—C6—C7	119.1 (2)	C24—C23—C22	121.1 (3)
C5—C6—C11	119.9 (2)	C24—C23—H23	119.5
C7—C6—C11	121.0 (2)	C22—C23—H23	119.5
O4—C7—C6	122.3 (2)	C23—C24—C25	123.0 (3)
O4—C7—C8	120.1 (2)	C23—C24—H24	118.5
C6—C7—C8	117.6 (3)	C25—C24—H24	118.5
C9—C8—C7	120.8 (3)	C30—C25—C26	119.5 (3)
C9—C8—H8A	119.6	C30—C25—C24	121.4 (3)
C7—C8—H8A	119.6	C26—C25—C24	119.1 (3)
C8—C9—C10	123.5 (3)	C27—C26—C25	117.3 (3)
C8—C9—H9	118.2	C27—C26—C21	124.5 (2)
C10—C9—H9	118.2	C25—C26—C21	118.2 (2)
C15—C10—C11	119.7 (3)	C28—C27—C26	121.7 (3)
C15—C10—C9	121.9 (3)	C28—C27—H27	119.2
C11—C10—C9	118.4 (3)	C26—C27—H27	119.2
C12—C11—C10	116.9 (3)	C27—C28—C29	120.7 (3)
C12—C11—C6	124.4 (2)	C27—C28—H28	119.7
C10—C11—C6	118.6 (2)	C29—C28—H28	119.7
C13—C12—C11	121.9 (3)	C30—C29—C28	119.2 (3)
C13—C12—H12	119.1	C30—C29—H29	120.4
C11—C12—H12	119.1	C28—C29—H29	120.4
C12—C13—C14	120.8 (3)	C29—C30—C25	121.6 (3)
C12—C13—H13	119.6	C29—C30—H30	119.2
C14—C13—H13	119.6	C25—C30—H30	119.2
			
C5—N1—C4—C2	152.3 (2)	C20—N2—C19—C16	−154.9 (2)
C5—N1—C4—C1	−88.3 (3)	C20—N2—C19—C17	85.9 (3)
C5—N1—C4—C3	32.4 (3)	C20—N2—C19—C18	−37.1 (3)
O2—C2—C4—N1	56.2 (3)	O5—C16—C19—N2	−55.8 (3)
O2—C2—C4—C1	−60.9 (3)	O5—C16—C19—C17	59.7 (3)
O2—C2—C4—C3	177.73 (19)	O5—C16—C19—C18	−176.2 (2)
O1—C1—C4—N1	−179.4 (2)	O6—C17—C19—N2	−179.39 (19)
O1—C1—C4—C2	−62.6 (3)	O6—C17—C19—C16	64.8 (3)
O1—C1—C4—C3	58.5 (3)	O6—C17—C19—C18	−56.8 (3)
O3—C3—C4—N1	45.6 (3)	O7—C18—C19—N2	71.4 (3)
O3—C3—C4—C2	−72.8 (3)	O7—C18—C19—C16	−171.6 (2)
O3—C3—C4—C1	165.1 (2)	O7—C18—C19—C17	−47.9 (3)
C4—N1—C5—C6	179.3 (2)	C19—N2—C20—C21	−177.7 (2)
N1—C5—C6—C7	1.3 (4)	N2—C20—C21—C22	−0.2 (4)
N1—C5—C6—C11	−178.4 (2)	N2—C20—C21—C26	179.0 (2)
C5—C6—C7—O4	−1.3 (4)	C20—C21—C22—O8	0.8 (4)
C11—C6—C7—O4	178.4 (2)	C26—C21—C22—O8	−178.4 (2)
C5—C6—C7—C8	178.6 (2)	C20—C21—C22—C23	−179.1 (2)
C11—C6—C7—C8	−1.6 (4)	C26—C21—C22—C23	1.6 (4)
O4—C7—C8—C9	−177.6 (3)	O8—C22—C23—C24	177.6 (3)
C6—C7—C8—C9	2.5 (4)	C21—C22—C23—C24	−2.4 (4)
C7—C8—C9—C10	−0.7 (5)	C22—C23—C24—C25	0.1 (5)
C8—C9—C10—C15	−179.8 (3)	C23—C24—C25—C30	−178.5 (3)
C8—C9—C10—C11	−2.0 (5)	C23—C24—C25—C26	3.0 (5)
C15—C10—C11—C12	0.6 (4)	C30—C25—C26—C27	−1.3 (4)
C9—C10—C11—C12	−177.2 (3)	C24—C25—C26—C27	177.2 (3)
C15—C10—C11—C6	−179.4 (2)	C30—C25—C26—C21	177.8 (2)
C9—C10—C11—C6	2.8 (4)	C24—C25—C26—C21	−3.6 (4)
C5—C6—C11—C12	−1.2 (4)	C20—C21—C26—C27	1.2 (4)
C7—C6—C11—C12	179.0 (3)	C22—C21—C26—C27	−179.6 (3)
C5—C6—C11—C10	178.7 (2)	C20—C21—C26—C25	−177.9 (2)
C7—C6—C11—C10	−1.0 (4)	C22—C21—C26—C25	1.4 (4)
C10—C11—C12—C13	−0.7 (4)	C25—C26—C27—C28	0.9 (4)
C6—C11—C12—C13	179.3 (3)	C21—C26—C27—C28	−178.2 (3)
C11—C12—C13—C14	0.0 (5)	C26—C27—C28—C29	0.3 (5)
C12—C13—C14—C15	0.7 (5)	C27—C28—C29—C30	−1.1 (5)
C13—C14—C15—C10	−0.8 (5)	C28—C29—C30—C25	0.7 (5)
C11—C10—C15—C14	0.1 (4)	C26—C25—C30—C29	0.5 (5)
C9—C10—C15—C14	177.9 (3)	C24—C25—C30—C29	−178.0 (3)

### Hydrogen-bond geometry (Å, º)

**Table:**

*D*—H···*A*	*D*—H	H···*A*	*D*···*A*	*D*—H···*A*
N1—H1···O4	0.86	1.91	2.587 (3)	135
N2—H2···O8	0.86	1.89	2.575 (2)	135
O1—H1*C*···O5^i^	0.82	1.90	2.715 (3)	172
O2—H2*C*···O8^ii^	0.82	1.77	2.589 (3)	173
O3—H3···O6^iii^	0.82	1.91	2.706 (3)	163
O5—H5···O4^iv^	0.82	1.84	2.650 (2)	171
O6—H6···O2^v^	0.82	1.81	2.609 (2)	163
O7—H7···O3^vi^	0.82	2.19	2.972 (2)	159

Symmetry codes: (i) −*x*+2, *y*−1/2, −*z*+3/2; (ii) −*x*+1, *y*−1/2, −*z*+3/2; (iii) *x*−1, *y*, *z*; (iv) −*x*+1, *y*+1/2, −*z*+3/2; (v) −*x*+2, *y*+1/2, −*z*+3/2; (vi) *x*+1, *y*, *z*.
