# Supplementary figures and images for: A new polymorph of 1-({[1,3-dihy­droxy-2-(hy­droxy­meth­yl)propan-2-yl]iminio}meth­yl)naphthalen-2-olate
Source: Acta Crystallogr E Crystallogr Commun. 2015 Aug 26;71(Pt 9):o686–7. doi: 10.1107/S205698901501539X (PMC4555412; doi:10.1107/S205698901501539X)

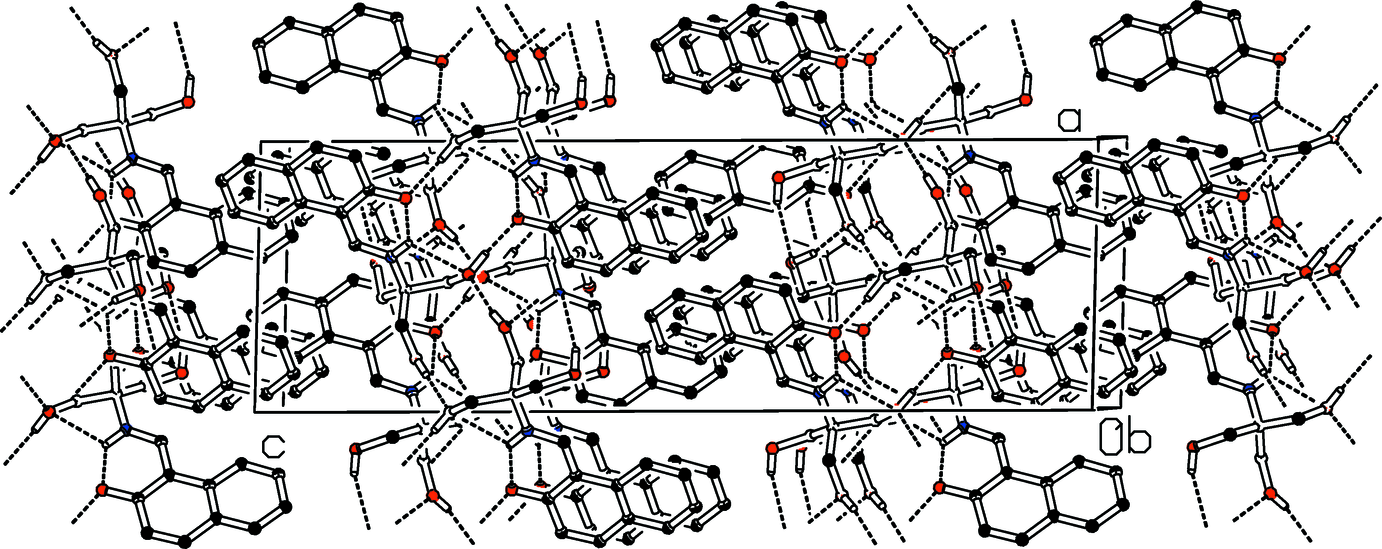

Supplement: Supplementary file 4 [file e-71-0o686-fig2.tif]
